# 18 F-FDG PET/CT Derived Volumetric Parameters Can Predict Outcome in Pediatric Ewing Sarcoma

**DOI:** 10.1007/s12149-026-02229-6

**Published:** 2026-05-21

**Authors:** Başak Soydaş-Turan, Bilge Volkan-Salancı, Burça Aydın, Pınar Kiratli

**Affiliations:** 1 Department of Nuclear Medicine, Kastamonu Education and Research Hospital, Kastamonu, Türkiye; 2 Faculty of Medicine, Department of Nuclear Medicine, Hacettepe University, Ankara, Türkiye; 3 Faculty of Medicine, Department of Pediatric Oncology, Hacettepe University, Ankara, Türkiye

**Keywords:** 18F-FDG PET/CT, Pediatric, Ewing sarcoma, Survival, Histopathologic response, Metabolic, Volumetric

## Abstract

**Objective:**

To evaluate the prognostic and predictive value of metabolic and volumetric parameters obtained from baseline (PET1) and post-chemotherapy (PET2) 18 F-FDG PET/CT in pediatric Ewing sarcoma (EWS) patients, focusing on their associations with histopathologic response, metastatic presentation, overall survival (OS), and progression-free survival (PFS).

**Methods:**

This retrospective single tertiary academic center study included 55 pediatric EWS patients who underwent PET1. Twenty-four patients also had PET2. SUVmax, SUVpeak, metabolic tumor volume (MTV), total lesion glycolysis (TLG) and their percentage changes (∆) between PET scans were calculated using absolute (SUV 2.0 and 2.5) and relative (40% and 45%) thresholds. Anatomic tumor volume was also measured. Histopathologic evaluation was available for 40 patients with PET1 and 18 patients with PET2. Associations were analyzed using logistic and Cox regression models.

**Results:**

Baseline metabolic and volumetric PET parameters were significantly higher in patients with presenting metastasis. Among these, TLG (45%) was identified as the only independent predictor of metastatic presentation (OR = 1.026, *p* = 0.019). Baseline MTV (2.0) was independently associated with shorter OS (HR = 1.026, *p* = 0.003) and PFS (HR = 1.035, *p* = 0.038). Combined stratification using MTV (2.0) and histologic response revealed that patients with an unfavorable histologic response but low MTV demonstrated superior OS and PFS rates than those with an unfavorable response and high MTV. The survival outcomes of patients with a favorable histologic response and high MTV were comparable to those with an unfavorable response and low MTV. However, PET parameters did not reliably predict histopathologic response in this cohort.

**Conclusions:**

Baseline MTV (2.0) demonstrated prognostic value for OS and PFS, while TLG (45%) independently predicted metastatic presentation in pediatric EWS. The combination of MTV (2.0) and histologic response appears to provide complementary prognostic information. Prospective, multicenter studies with standardized imaging and treatment protocols are warranted to validate the role of volumetric 18 F-FDG PET parameters in clinical risk stratification.

**Supplementary Information:**

The online version contains supplementary material available at 10.1007/s12149-026-02229-6.

## Introduction

Ewing sarcoma (EWS) is the second most common malignant bone tumor in children and adolescents, accounting for approximately 3% of pediatric cancers [1]. Classic prognostic factors include age, tumor size, tumor volume, primary site, metastatic disease, and histopathologic response [1–3]. Regardless of these parameters, survival outcomes remain inferior to those observed in lymphomas. Therefore, there is a need to improve risk stratification [4].

Histopathologic response to neoadjuvant chemotherapy is widely regarded as an important indicator of long-term outcome [5]. Nevertheless, it can be assessed only after surgical resection, limiting its utility for early treatment adaptation, and is unavailable for a substantial portion of patients (approximately 20–40%) who are treated with radiation only [6]. This situation has driven the search for non-invasive biomarkers that can provide clinically relevant prognostic information earlier in the disease course.

18 F-FDG PET/CT has an important role in staging of primary and recurrent EWS [7–9]; however, its role in quantitative risk stratification remains less clearly defined. PET-derived metabolic and volumetric parameters such as maximum or peak standardized uptake value (SUV), metabolic tumor volume (MTV), and total lesion glycolysis (TLG) may provide additional insights into tumor aggressiveness. Nevertheless, prior studies assessing the predictive and prognostic value of these parameters have reported inconsistent findings, which may reflect differences in cohort composition [10–16] and non-uniform treatment protocols [11]. On the other hand, adoption of volumetric PET parameters has been limited by the lack of standardized calculation methods and inconsistent results across studies. Moreover, only a few reports have examined associations between PET-derived volumetric parameters and survival specifically in pediatric EWS [15, 16], whereas most prior work had focused primarily on the relationship between SUVmax and histopathologic response.

In this EWS study focusing on pediatric population, our aim was to use different threshold methods for volumetric PET parameters and examine their relationship with metastatic presentation and survival rather than histopathological response alone, while exploring whether survival and histologic response could be predicted at an earlier stage using 18 F-FDG PET imaging.

## Materials and methods

### Patients and data collection

This retrospective study included pediatric EWS patients who undergone 18 F-FDG PET/CT for initial staging (PET1) at Hacettepe University between January 2012 and December 2020. If 18 F-FDG PET/CT was performed prior to local tumor control after neoadjuvant chemotherapy, these scans were also included (PET2). A total of 55 pediatric EWS patients who had PET1 scan were included in this study, of whom 24 also had PET2 scan.

Clinical data, including age at diagnosis, gender, tumor site and size, presence of metastasis at diagnosis, treatment modalities, histopathologic response and follow-up information were obtained from medical records. Patients were considered to have a favorable histopathologic response when less than 10% viable tumor cells were identified on pathology report.

18 F-FDG PET/CT studies were obtained from the institutional PACS and re-evaluated.

This study was performed in line with the principles of the Declaration of Helsinki. Approval was granted by the Hacettepe University Ethics Committee (Approval No: GO 22/230).

### Image analysis

Image analysis was performed retrospectively using both visual assessment and semi-quantitative measurements by two nuclear medicine physicians (BST and BVS) blinded to clinical outcomes, on a dedicated workstation (GE Healthcare AW4.6 Chicago, IL, USA). For semi-quantitative analysis, a spherical volume of interest (VOI) encompassing the entire primary tumor was initially delineated by BST and subsequently reviewed by BVS. In cases of discrepancy, a third nuclear medicine physician (POK) provided the final decision. Quantitative PET parameters including SUVmax, SUVpeak, MTV and TLG were derived from the primary tumor. MTV was obtained through threshold-based segmentation using both absolute SUV (2.0 and 2.5) and relative (40% and 45% of SUVmax) thresholds. TLG was calculated by multiplying MTV by SUVmean. Corresponding MTV and TLG values were reported according to the applied threshold.

Absolute thresholds of 2.0 and 2.5 were chosen for MTV calculation based on a previous study showing that a lower threshold (e.g., 1.5) cannot clearly distinguish the tumor from adjacent normal tissue, whereas a higher threshold (e.g., 3.0) may underestimate tumor volume [17]. For relative threshold, 40% and 45% of SUVmax were selected, as higher percentages (e.g., 60%) tend to underestimate true tumor volume on initial scans, particularly in tumors with high SUVmax [18]. These thresholds inherently exclude low-grade peripheral uptake and minimize the potential influence of post-biopsy inflammatory uptake for PET1 scans. In fact, no patient demonstrated intense inflammatory uptake that mimicked or exceeded the metabolic activity of the primary tumor.

Changes in PET-derived parameters between PET1 and PET2 scans were expressed as percentage differences, calculated using the formula: ∆PET = 100 × (PET2 – PET1) / PET1. Anatomic tumor volume was calculated from magnetic resonance imaging (MRI) performed within 2 weeks of 18 F-FDG PET/CT, using the ellipsoid formula (π/6 × length × depth × width) [19].

Metastatic disease was defined using a combination of metabolic and morphological criteria. Any focal 18 F-FDG uptake above background activity that could not be explained by physiological or clinical conditions was considered suspicious for metastasis. Suspected skeletal lesions were further evaluated and confirmed with MRI. All patients underwent chest CT to assess for possible pulmonary metastases. Lymph nodes with 18 F-FDG uptake were assessed by ultrasound, and nodes demonstrating benign or reactive features were not considered metastatic. Lesions showing clear progression in number or size on follow-up examinations were also regarded as metastatic. Histopathologic confirmation was obtained when clinically indicated.

### Statistical analysis

Statistical analyses were performed using SPSS v23.0 (IBM, NY, USA) and p-value < 0.05 was used for statistical significance. Continuous variables were summarized as mean ± standard deviation for normally distributed data or as median (minimum – maximum) for non-normally distributed data, and categorical variables were expressed as frequencies and percentages. Normality was tested using the Kolmogorov-Smirnov test.

Comparisons of continuous variables between two independent groups were performed using the Mann-Whitney U-test. If the variables were categorical, the Pearson chi-square test was used.

The predictive ability of clinical and imaging parameters for the presence of metastasis and histopathological response was evaluated using binary logistic regression analysis, while their effects on overall survival (OS) and progression-free survival (PFS) were assessed using Cox regression analysis. OS was calculated from the initiation of chemotherapy to death. PFS was defined as the time from initiation of chemotherapy to radiologic disease progression/relapse. Patients without an event were censored at the date of last clinical contact. Initially, univariate analyses were performed for binary logistic and Cox regression analyses. For clinical interpretation, optimal cut-off values of significant continuous variables were determined and presented based on the maximum Youden’s index. PET parameters with *p* < 0.05 in univariate logistic or Cox regression analyses were considered for inclusion in multivariate models. To avoid multicollinearity, pairwise correlations between PET parameters were assessed using Spearman’s correlation test. When a strong correlation was observed (*r* > 0.8), the PET parameter with the highest area under the curve (AUC) on receiver-operating characteristic (ROC) analysis was selected and included in the multivariate model using backward elimination together with relevant clinical parameters. In logistic and Cox regression analyses, MTV and TLG values were initially assessed as increases per 1-unit. However, expressing the odds/hazard ratio per 1-unit increase was found to represent very small percentage changes. Therefore, to improve clinical interpretability, odds/hazard ratios were expressed per 10-unit increase (for example, a 10 mL increase for MTV or a 10-point numerical increase for TLG). For this purpose, the original MTV and TLG values were divided by 10. Since the odds/hazard ratios were close to 1, expressing the results as percentage increases [(odds/hazard ratio – 1) × 100%] was considered to provide a clearer interpretation than stating that the risk increased by a certain fold. In addition to these, Kaplan–Meier curves were generated and differences between groups defined by the Youden index–based cut-off values were assessed using the log-rank test.

## Results

### Patients’ characteristics and treatment protocols

A total of 55 pediatric EWS patients who had baseline 18 F-FDG PET/CT (PET1) were included in this study. The mean age at diagnosis was 10 years. Metastatic disease presentation was observed in 23 patients (41.8%). A subset of 24 patients also had 18 F-FDG PET/CT after chemotherapy (PET2). The clinical characteristics are summarized in Table 1.

**Table 1:** Patients’ characteristics

Variables	
**Age** (mean ± standard deviation)	10.3 ± 3.5 years
**Largest tumor size** [median (min-max)]	8.2 (2–25) cm
**Anatomic tumor volume** [median (min-max)]	86.9 (3.6-3556.4) mL
	**N (%)**
**Female**	22 (40)
**Primary tumor site**	
Appendicular	31 (56.4)
Axial	19 (34.5)
Soft tissue	5 (9.1)
**Metastasis at diagnosis**	23 (41.8)
Bone	12 (21.8)
Lung	8 (14.5)
Lymph nodes	8 (14.5)
**Histopathologic response**	
Favorable (≤ 10% viable tumor)	30 (54.5)
Unfavorable (> 10% viable tumor)	10 (18.2)
Not applicable*	15 (27.3)

*includes patients who did not undergo surgical resection or whose pathology reports did not contain evaluable necrosis percentage

All patients received neoadjuvant chemotherapy. Most patients (*n* = 42) were treated with the EuroEwing 99 regimen. Surgical resection of the primary tumor was performed in 46 patients. Six patients received only radiotherapy for local control. Patients received adjuvant chemotherapy following local control. In cases of relapse, patients were re-treated with chemotherapy, and additional surgery or radiotherapy was performed whenever feasible to achieve disease control. The details of treatment protocols were summarized in Supplementary Table 1.

The median follow-up time after diagnosis was not reached using the reverse Kaplan–Meier method; the mean follow-up was 108.7 months (95% CI = 92.1–125.3). During follow-up, disease relapse or progression was observed in 22 patients (40%). Relapse status was unknown for 1 patient who was lost to follow-up. Overall, 19 patients (34.5%) died (one patient due to acute myeloid leukemia).

### Association between 18 F-FDG PET parameters and histopathological response

Histopathological response data were available for 40 patients with PET1 and 18 patients with PET2. There were no significant differences in SUVmax, SUVpeak, MTV, or TLG values obtained from PET1 and PET2 between patients with favorable and unfavorable histopathological responses. Similarly, the percentage changes in these parameters (ΔSUVmax, ΔSUVpeak, ΔMTV and ΔTLG) did not differ significantly between the two groups (*p* > 0.05 for all, Supplementary Table 2). Also, logistic regression analysis revealed no independent association between any of the PET-derived parameters and histopathological response (not shown).

### Relationship between 18 F-FDG PET parameters and metastatic presentation

Patients with metastatic presentation exhibited higher baseline SUVmax, SUVpeak, MTV, and TLG values, as well as larger anatomic tumor volumes than those with localized disease (Table 2).

**Table 2:** Comparison of PET1 parameters between metastatic and localized disease groups

Variables	Metastatic Disease	Localized Disease	*p*
Largest tumor size	8.8 (3.2–20)	7.3 (2–25)	0.107
Anatomic tumor volume	190.3 (23.4-1936.8)	59.8 (3.5-3556.4)	**0.001**
SUVmax	7.1 (1.6–19.5)	4.9 (1.7–22.7)	**0.018**
SUVpeak	5.9 (1.3–17.3)	3.9 (1.4–18.7)	**0.012**
MTV (40%)	144 (33.3–1022)	41.5 (2.9–1264)	**< 0.001**
MTV (45%)	107 (23.4–848)	35.3 (2.5–962)	**< 0.001**
MTV (2.0)	184 (0-1824)	35.2 (0-800)	**< 0.001**
MTV (2.5)	148.4 (0-1665)	14.7 (0-710)	**0.001**
TLG (40%)	584.8 (69.4-7324.7)	124.2 (6.9-2138.1)	**< 0.001**
TLG (45%)	473.4 (52.6-6364.5)	100.7 (6.3-1687.4)	**< 0.001**
TLG (2.0)	771.7 (0-10205.6)	96.5 (0-3999.9)	**< 0.001**
TLG (2.5)	636.6 (0-9839)	53.9 (0-3898.7)	**0.001**

Variables are presented as median (min-max) values. Bold p-values represent statistically significant results. PET1: baseline 18 F-FDG PET/CT, MTV: metabolic tumor volume, TLG: total lesion glycolysis

In univariate logistic regression (Table 3), female patients and patients with axial tumors tended to have a higher likelihood of metastatic disease. Among PET parameters, higher SUVpeak, MTV (2.0), TLG (40%), and TLG (45%) were significantly associated with metastatic disease. Each unit increase in SUVpeak corresponded to a 20% higher likelihood of metastasis. Similarly, for each 10-unit increase in MTV (2.0) and TLG values, particularly TLG (40% and 45%), the probability of metastasis increased by approximately 4% and 2%, respectively. Notably, tumor size and anatomic tumor volume showed no significant association with metastatic presentation.

**Table 3:** Results of univariate and multivariate logistic regression analyses for presence of metastasis at diagnosis

Variables		Categories or Cut-offs	OR (95% CI)	*p*
**Univariate**				
Age		Per unit	1.02 (0.87–1.19)	0.756
Gender		Female vs. Male	2.83 (0.88–9.04)	0.079
Tumor site		Soft tissue vs. Axial vs. Append.		0.019
Soft tissue (reference)				
Axial			8.66 (0.79–95.08)	0.077
Appendicular			1.63 (0.16–16.72)	0.678
Largest tumor size		Per unit	1.09 (0.96–1.24)	0.164
Anatomic tumor volume		Per 10-unit	1.003 (0.993–1.014)	0.529
**PET1-derived parameters**				
SUVmax		Per unit	1.16 (0.99–1.36)	0.066
SUVpeak		Per unit	1.204 (1.001–1.449)	**0.049**
SUVpeak		≤ 5.93 vs. > 5.93	6.76 (1.91–23.91)	**0.003**
MTV (40%)		Per 10-unit	1.031 (0.996–1.067)	0.086
MTV (45%)		Per 10-unit	1.044 (0.996–1.093)	0.072
MTV (2.0)		Per 10-unit	1.042 (1.004–1.081)	**0.028**
MTV (2.0)		≤ 61.71 vs. > 61.71	16.96 (4.33–66.47)	**< 0.001**
MTV (2.5)		Per 10-unit	1.040 (0.999–1.082)	0.054
TLG (40%)		Per 10-unit	1.017 (1.002–1.032)	**0.031**
TLG (40%)		≤ 303.25 vs. > 303.25	19.44 (4.91–76.93)	**< 0.001**
TLG (45%)		Per 10-unit	1.023 (1.003–1.043)	**0.024**
TLG (45%)		≤ 227 vs. > 227	20.58 (5.09–83.18)	**< 0.001**
TLG (2.0)		Per 10-unit	1.006 (1.000-1.012)	0.063
TLG (2.5)		Per 10-unit	1.005 (0.999–1.011)	0.083
**Multivariate (backward elimination)**				
TLG (45%)		Per 10-unit	1.026 (1.004–1.048)	**0.019**
Tumor site		Soft tissue vs. Axial vs. Append.		0.028
Soft tissue (reference)				
Axial			57.18 (0.83-3907.23)	0.060
Appendicular			11.76 (0.17-788.01)	0.250

Bold p-values represent statistically significant results. Continuous variables were analyzed per one-unit (a 1-point numerical increase for SUV parameters) or 10-unit increase (a 10 mL increase for MTV and a 10-point numerical increase for TLG). For clinical interpretation, optimal cut-off values of statistically significant continuous variables were determined based on the maximum Youden’s index. Gender, tumor site, SUVpeak, and TLG (45%) were entered as independent variables into the multivariate model. MTV (2.0) was not included because it showed strong correlations with both SUVpeak and TLG (45%). OR: odds ratio, CI: confidence interval, Append: appendicular, PET1: baseline 18 F-FDG PET/CT, MTV: metabolic tumor volume, TLG: total lesion glycolysis

To identify independent predictors, SUVpeak and TLG (45%) were entered into the multivariate model together with gender and primary tumor site. TLG (45%) was selected instead of TLG (40%) because it demonstrated a slightly higher AUC value in ROC analysis (AUC = 0.834, *p* < 0.001 vs. AUC = 0.833, *p* < 0.001). MTV (2.0) was not included in the model due to its strong correlations with both SUVpeak and TLG metrics (*r* > 0.8), as well as its relatively lower AUC compared with TLG (45%) [AUC = 0.810, *p* < 0.001 vs. AUC = 0.834, *p* < 0.001].

In the final model, TLG (45%) remained the independent predictor of metastatic disease at diagnosis (OR = 1.026 per 10-unit increase, *p* = 0.019). Notably, the same result was obtained when MTV (2.0), which was not included initially due to its strong correlations, was entered into the same model.

### Survival analysis

The mean OS and PFS for the cohort were 109 and 89 months, respectively. The median OS and PFS has not been reached. One patient who died due to secondary acute myeloid leukemia was excluded from the OS analysis. One patient who was lost to follow-up after neoadjuvant chemotherapy until notification of death was excluded from the PFS analysis.

### Overall survival

In univariate Cox regression (Table 4), metastasis at diagnosis was significantly associated with inferior OS. Among PET1-derived parameters, MTV (2.0), MTV (2.5), and all TLG parameters showed significant associations with OS, whereas SUVmax and SUVpeak demonstrated near-significance. Each 10-unit increase in MTV (2.0) and MTV (2.5) was associated with approximately a 4% higher risk of death. Each 10-unit increase in TLG values, the hazard of death increased by 0.4% to 0.8%.

**Table 4:** Results of univariate and multivariate Cox regression analyses for overall survival

Variables		Categories or Cut-offs	HR (95% CI)	*p*
**Univariate**				
Age		Per unit	1.08 (0.95–1.23)	0.212
Gender		Female vs. Male	1.43 (0.53–3.38)	0.471
Tumor site		Soft tissue vs. Axial vs. Append.		0.271
Soft tissue (reference)				
Axial			3.26 (0.40-26.37)	0.267
Appendicular			1.64 (0.20-13.03)	0.639
Metastasis at diagnosis		Localized vs. Metastatic	2.68 (1.04–6.93)	**0.041**
Histopathological response		Favorable vs. Unfavorable	3.14 (0.98–10.03)	0.053
Anatomic tumor volume		Per 10-unit	1.001 (0.993–1.008)	0.842
**PET1-derived parameters**				
SUVmax		Per unit	1.08 (0.99–1.17)	0.064
SUVmax		≤ 9.21 vs. > 9.21	4.72 (1.73–12.89)	**0.002**
SUVpeak		Per unit	1.09 (0.99–1.21)	0.061
SUVpeak		≤ 7.77 vs. > 7.77	4.09 (1.49–11.20)	**0.006**
MTV (40%)		Per 10-unit	1.011 (0.998–1.025)	0.103
MTV (45%)		Per 10-unit	1.015 (0.997–1.033)	0.100
MTV (2.0)		Per 10-unit	1.039 (1.021–1.056)	**< 0.001**
MTV (2.0)		≤ 48.75 vs. > 48.75	4.42 (1.28–15.32)	**0.019**
MTV (2.5)		Per 10-unit	1.037 (1.017–1.057)	**< 0.001**
MTV (2.5)		≤ 21.51 vs. > 21.51	3.01 (0.97–9.28)	0.056
TLG (40%)		Per 10-unit	1.007 (1.004–1.011)	**< 0.001**
TLG (40%)		≤ 145.10 vs. > 145.10	4.13 (1.19–14.29)	**0.025**
TLG (45%)		Per 10-unit	1.008 (1.004–1.012)	**< 0.001**
TLG (45%)		≤ 130.35 vs. > 130.35	4.13 (1.19–14.29)	**0.025**
TLG (2.0)		Per 10-unit	1.004 (1.002–1.007)	**0.001**
TLG (2.0)		≤ 121.75 vs. > 121.75	3.98 (1.15–13.80)	**0.029**
TLG (2.5)		Per 10-unit	1.004 (1.002–1.007)	**0.002**
TLG (2.5)		≤ 248.30 vs. > 248.30	2.70 (1.02–7.13)	**0.045**
**Multivariate (backward elimination)**				
MTV (2.0)		Per 10-unit	1.039 (1.021–1.056)	**< 0.001**

Bold p-values represent statistically significant results. Continuous variables were analyzed per one-unit (a 1-point numerical increase for SUV parameters) or 10-unit increase (a 10 mL increase for MTV and a 10-point numerical increase for TLG). Optimal cut-off values of statistically significant continuous variables were determined based on the maximum Youden’s index. To avoid multicollinearity, MTV (2.0), which demonstrated the highest AUC in ROC analysis, was selected for inclusion in the multivariate model together with relevant clinical variables. HR: hazard ratio, CI: confidence interval, Append: appendicular, PET1: baseline 18 F-FDG PET/CT, MTV: metabolic tumor volume, TLG: total lesion glycolysis

Because these PET parameters were strongly intercorrelated (*r* > 0.8), MTV (2.0), which demonstrated the highest predictive performance for OS in ROC analysis (AUC = 0.725, *p* = 0.007), was selected for inclusion in the multivariate model together with relevant clinical variables, including age, gender, tumor site, presence of metastasis, and anatomic tumor volume. MTV (2.0) remained an independent predictor of OS (HR = 1.039 per 10-unit increase, *p* < 0.001).

**Fig. 1:**
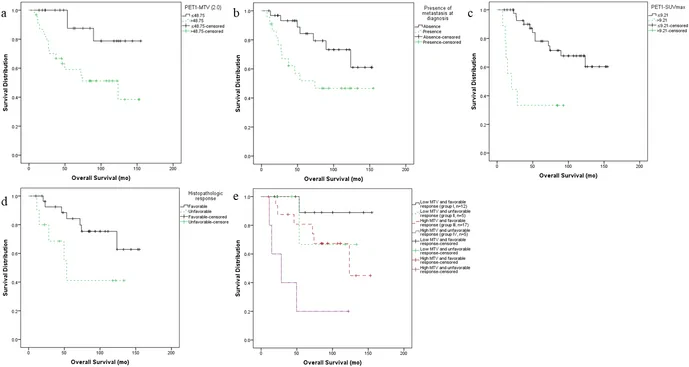
Kaplan–Meier survival curves for overall survival (OS) according to clinical and PET-derived parameters in pediatric EWS. (**a**) Patients with higher baseline metabolic tumor volume [MTV (2.0) > 48.75] showed reduced OS compared with those below the cut-off (estimated 5-year OS: 59% vs. 87%, *p* = 0.010). (**b**) Metastatic presentation was linked to decreased OS (estimated 5-year OS: 52% vs. 84%, *p* = 0.034). (**c**) Higher baseline SUVmax (> 9.21) corresponded to shorter OS (estimated 5-year OS: 33% vs. 78%, *p* = 0.001). (**d**) Unfavorable histopathologic response was associated with lower OS compared with favorable response (estimated 5-year OS: 41% vs. 84%, *p* = 0.041). (**e**) OS curves of risk groups defined by MTV (2.0) and histopathologic response: 10-year OS rates for group I-IV were 88.9%, 66.7%, 67.3%, and 20%, respectively

Kaplan–Meier analysis showed significantly poorer OS in patients with baseline MTV (2.0) > 48.75 (Fig. 1a), those presenting with metastatic disease at diagnosis (Fig. 1b), patients with baseline SUVmax > 9.21 (Fig. 1c), and those with an unfavorable histopathologic response (Fig. 1d).

Histopathologic response, which showed a near-significant association in univariate analysis, was not included in the multivariate model because it was available for only a subset of patients (72%). However, to further explore its effect with baseline MTV (2.0) on OS, the cohort was divided into four groups: group I, patients with MTV (2.0) ≤ 48.75 and a favorable response (*n* = 12); group II, patients with MTV (2.0) ≤ 48.75 and an unfavorable response (*n* = 5); group III, patients with MTV (2.0) > 48.75 and a favorable response (*n* = 17); and group IV, patients with MTV(2.0) > 48.75 and an unfavorable response (*n* = 5). The corresponding 10-year OS rates for group I-IV were 88.9%, 66.7%, 67.3%, and 20%, respectively [Fig. 1e]. Among patients with unfavorable responses, those with low MTV (2.0) values tended to have better median OS (not reached vs. 28 months, *p* = 0.068). Among those with favorable responses, higher MTV (2.0) values were associated with slightly poorer median survival (123 months vs. not reached), without statistical significance (*p* = 0.138). Interestingly, the prognosis of patients with a favorable histopathologic response but high MTV was comparable to that of patients with an unfavorable histopathologic response but low MTV (*p* = 0.715).

None of the metabolic and volumetric parameters obtained from PET2 (*n* = 23), nor any of the ΔPET parameters, demonstrated prognostic significance for OS.

### Progression-free survival

In univariate analysis (Table 5), higher baseline SUVmax, SUVpeak and MTV (2.0) were significantly associated with shorter PFS. Each unit increase in SUVmax and SUVpeak corresponded to an 8.5% and 9.6% higher risk of disease progression, respectively. Likewise, for each 10-unit increase in MTV (2.0), the risk of progression increased by approximately 1%.

**Table 5:** Results of univariate and multivariate Cox regression analyses for progression-free survival

Variables		Categories or Cut-offs	HR (95% CI)	*p*
**Univariate**				
Age		Per unit	1.02 (0.91–1.15)	0.655
Gender		Female vs. Male	1.08 (0.46–2.53)	0.859
Tumor site		Soft tissue vs. Axial vs. Append.		0.501
Soft tissue (reference)				
Axial			1.56 (0.33–7.29)	0.570
Appendicular			0.92 (0.20–4.19)	0.921
Metastasis at diagnosis		Localized vs. Metastatic	1.90 (0.82–4.40)	0.133
Histopathological response		Favorable vs. Unfavorable	2.38 (0.79–7.17)	0.121
Anatomic tumor volume		Per 10-unit	0.999 (0.992–1.007)	0.889
**PET1-derived parameters**				
SUVmax		Per unit	1.085 (1.007–1.170)	**0.033**
SUVmax		≤ 10.72 vs. > 10.72	6.39 (2.40-16.97)	**< 0.001**
SUVpeak		Per unit	1.096 (1.001–1.199)	**0.046**
SUVpeak		≤ 7.77 vs. > 7.77	2.86 (1.16–7.07)	**0.022**
MTV (40%)		Per 10-unit	1.004 (0.989–1.019)	0.607
MTV (45%)		Per 10-unit	1.004 (0.985–1.024)	0.671
MTV (2.0)		Per 10-unit	1.010 (1.001–1.019)	**0.031**
MTV (2.0)		≤ 224 vs. > 224	4.08 (1.69–9.85)	**0.002**
MTV (2.5)		Per 10-unit	1.009 (0.998–1.020)	0.094
TLG (40%)		Per 10-unit	1.002 (1.000-1.004)	0.116
TLG (45%)		Per 10-unit	1.002 (0.999–1.005)	0.163
TLG (2.0)		Per 10-unit	1.002 (1.000-1.003)	0.062
TLG (2.5)		Per 10-unit	1.002 (1.000-1.003)	0.091
**Multivariate (backward elimination)**				
MTV (2.0)		Per 10-unit	1.035 (1.002–1.070)	**0.038**

Bold p-values represent statistically significant results. Continuous variables were analyzed per one-unit (a 1-point numerical increase for SUV parameters) or 10-unit increase (a 10 mL increase for MTV and a 10-point numerical increase for TLG). Optimal cut-off values of statistically significant continuous variables were determined based on the maximum Youden’s index. To avoid multicollinearity, MTV (2.0), which demonstrated the highest AUC in ROC analysis, was selected for inclusion in the multivariate model together with relevant clinical variables. HR: hazard ratio, CI: confidence interval, Append: appendicular, PET1: baseline 18 F-FDG PET/CT, MTV: metabolic tumor volume, TLG: total lesion glycolysis

Because these PET parameters were found to be highly correlated with each other (*r* > 0.8), MTV (2.0), which demonstrated the highest AUC for relapse in ROC analysis (AUC = 0.635, *p* = 0.094), was incorporated in the multivariate model together with the relevant clinical variables including age, gender, tumor site, presence of metastasis at diagnosis, anatomic tumor volume. MTV (2.0) remained independently associated with shorter PFS (HR = 1.035 per 10-unit increase, *p* = 0.038). Of note, inclusion of SUVmax in the same model yielded similar results.

**Fig. 2:**
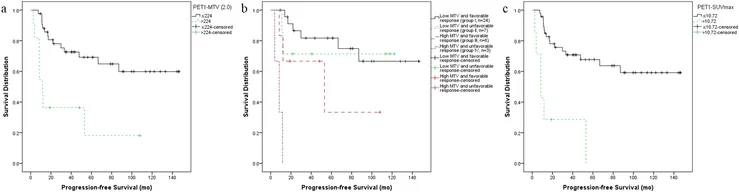
Kaplan–Meier curves for progression-free survival (PFS) based on clinical and PET-derived parameters in pediatric EWS. (**a**) Higher metabolic tumor volume [MTV (2.0) > 224] was associated with shorter PFS (estimated 5-year PFS: 18% vs. 69%, *p* = 0.001). (**b**) PFS curves of risk groups defined by MTV (2.0) and histopathologic response showed the difference in PFS among the 4 groups, except for between group I and group II (*p* = 0.730), group I and III (*p* = 0.091) and group II and group III (*p* = 0.489). (**c**) Higher baseline SUVmax (> 10.72) was associated with poorer PFS (median PFS: 8.5 months vs. not reached, *p* < 0.001)

Kaplan–Meier analysis demonstrated that significantly shorter PFS in patients with MTV (2.0) > 224 (estimated 5-year PFS: 18% vs. 69%, *p* = 0.001; Fig. 2a) compared to those with MTV (2.0) < 224.

To examine the impact of histopathologic response, the cohort was divided into four groups (Fig. 2b): group I, patients with MTV (2.0) ≤ 224 and a favorable response (*n* = 24); group II, patients with MTV (2.0) ≤ 224 and an unfavorable response (*n* = 7); group III, patients with MTV(2.0) > 224 and a favorable response (*n* = 6); and group IV, patients with MTV(2.0) > 224 and an unfavorable response (*n* = 3). Among patients with unfavorable responses, those with low MTV (2.0) values had significantly better median PFS compared to those with high MTV (2.0) values (not reached vs. 8 months, *p* = 0.005). Among patients with favorable responses, those with high MTV (2.0) values tended to have shorter median PFS (53 months vs. not reached; *p* = 0.091). Notably, patients with a favorable histopathologic response but high MTV had comparable outcome to those with an unfavorable response but low MTV (*p* = 0.489).

None of the SUVmax, SUVpeak, MTV, or TLG values obtained from PET2 (*n* = 24), nor any of the ΔPET parameters, demonstrated prognostic significance for PFS.

## Discussion

This study evaluated the predictive and prognostic value of 18 F-FDG PET parameters in pediatric EWS patients. The ability of 18 F-FDG PET to predict histopathological response in EWS remains uncertain. In a cohort of 36 patients that included both children and adults, the concordance between post-chemotherapy SUVmax ≤ 2.5 and histologic response was only 68% [11]. In our study, PET2 SUVmax (cut-off 2.4) showed similar suboptimal accuracy (72%, *p* = 0.245; which was not mentioned in the results due to lack of statistical significance). Importantly, no SUVmax threshold reliably predicted response in that study, and the chemotherapy regimens were heterogenous [11], as in our cohort. Likewise, a pediatric study of 50 uniformly treated patients failed to identify any absolute SUVmax cut-off value capable of predicting histologic response [20], and another investigation with mixed cohort using a post-chemotherapy SUVmax threshold of 3 also reported no clinically meaningful accuracy [10]. In contrast, in a pediatric work by El-Hennawy et al. [21], several post-chemotherapy PET parameters, including MTV (2.5) [cut-off 0.14], MTV (liver SUVmean) [cut-off 17], TLG (liver SUVmean) [cut-off 60], SUVmax (cut-off 2.5), and ΔTLG (liver SUVmean) [cut-off – 90%] were able to distinguish favorable from unfavorable responders. Similarly, Annovazzi et al. [10] showed in a mixed cohort that ΔTLG (40%) > – 60% was significantly associated with favorable histopathologic response.

Considering together, none of the published studies, including ours, have demonstrated a consistent association between baseline 18 F-FDG PET parameters and histopathological response in pediatric EWS. Although PET2 and ΔPET metrics did not reach statistical significance in our cohort, likely due to the limited number of evaluable patients, previous studies have suggested that post-chemotherapy 18 F-FDG PET may be more informative and may have potential clinical implications. For example, if 18 F-FDG PET-derived parameters obtained after chemotherapy suggest a poor histopathologic response, this information could theoretically be used to guide treatment intensification. We acknowledge that this concept remains speculative and requires validation in larger prospective studies. Moreover, recent literature increasingly highlights the potential importance of volumetric parameters such as MTV and TLG rather than SUVmax alone. Future studies may also benefit from incorporating radiomic features, which could provide additional insight beyond conventional PET metrics.

Our findings indicate that baseline metabolic and volumetric PET parameters were significantly higher in patients with metastatic presentation. Among these, TLG (45%) remained the only independent predictor of metastatic disease in multivariate analysis. Importantly, anatomic tumor volume did not show comparable predictive ability, highlighting the dominant role of PET parameters as a marker of aggressive tumor biology compared to structural measurements.

Several prior studies in EWS have proposed that 18 F-FDG PET parameters may have prognostic value for OS or PFS, with particular emphasis on SUVmax measured either at baseline [12–14, 22] or after neoadjuvant chemotherapy [10, 11, 20]. However, some pediatric studies found no association between SUVmax, MTV, or TLG and survival outcomes [15, 16], reflecting the heterogeneity and methodological variability across published data. In our study, a baseline SUVmax > 9.2 was associated with a nearly 5-fold increased risk of death, whereas a baseline SUVmax > 10.7 was associated with a nearly 6-fold higher risk of progression. On the other hand, baseline MTV (2.0) demonstrated the strongest predictive performance and remained an independent prognostic factor of OS and PFS in multivariate analysis.

Histopathologic response to neoadjuvant chemotherapy did not reach the statistical significance in the current series, probably due to low number of patients analyzed and heterogenous chemotherapy protocols. Nevertheless, further stratification combining MTV (2.0) and histologic response suggested that these two markers may provide complementary prognostic information for both OS and PFS. Patients with an unfavorable histologic response but low MTV demonstrated superior OS and PFS rates compared with those with an unfavorable response and high MTV. From a clinical perspective, it may suggest that, among patients with unfavorable histopathologic response, those with higher baseline MTV could be considered for more intensive treatment strategies compared to those with lower baseline MTV. Notably, the OS and PFS of patients with a favorable histologic response and high MTV were comparable to those of patients with an unfavorable response and low MTV, indicating that metabolic parameter may partially offset the prognostic effect of histologic necrosis alone. However, due to the small sample size in subgroups, these findings should be interpreted as hypothesis-generating.

None of the metabolic parameters derived from PET2 or ΔPET demonstrated prognostic significance for OS or PFS in our cohort. This stands in contrast to some prior reports. We reviewed the characteristics of the patients who underwent PET2 to evaluate the possibility of selection bias. Although the proportion of patients with metastatic disease at diagnosis (high-risk) was numerically higher in the PET2 group [13/24 (54%) vs. 10/31 (32%)], this difference did not reach statistical significance (*p* = 0.102). Moreover, PET2 was not exclusively performed in high-risk patients, as some patients without adverse prognostic features underwent PET2 (*n* = 8), while some with unfavorable characteristics (*n* = 16) did not. In addition, PET2 findings were consistent with a favorable metabolic response. When histopathological response was considered, the proportion of patients with unfavorable response was not higher in the PET2 group. These findings suggest that PET2 was not preferentially performed only in patients with high-risk disease or poor treatment response. However, some degree of selection bias cannot be completely excluded due to the retrospective design. Therefore, we believe that the lack of a significant association between PET2 parameters and prognosis or histopathological response is likely influenced not only by potential selection bias but also by the limited sample size.

A major strength of our study is that it is one of the largest pediatric EWS cohorts in which baseline 18 F-FDG PET parameters have been systematically evaluated. In addition, it compares volumetric PET parameters obtained using different thresholding methods. However, the retrospective and single-center design introduced certain inherent limitations. Most notably, the availability of PET2 scans and histopathologic response data was restricted to a subset of patients, limiting the statistical power of post-treatment analyses. The other limitation of our analysis is the heterogeneity of chemotherapy regimens. Variations in drug combinations and the number of chemotherapy cycles administered between PET1 and PET2 may have limited predictive performance of PET2. Although the retrospective design and treatment heterogeneity mandate cautious interpretation of our findings, we believe that the observed prognostic signals, particularly for baseline MTV (2.0), may be clinically meaningful; however external validation is required to ensure the generalizability of our findings. Prospective studies with standardized PET acquisition (e.g., imaging after a fixed number of comparable chemotherapy cycles) and processing protocols are needed to clarify the clinical role of 18 F-FDG PET in risk stratification.

## Conclusion

In this cohort, PET-derived parameters did not reliably predict histopathologic response. However, two important messages can be drawn from the present study. First, TLG (45%) was the strongest predictor of metastatic presentation at diagnosis. Second, we demonstrate in a relatively large pediatric cohort that baseline MTV (2.0) shows a potential prognostic association with both OS and PFS. This discrepancy likely reflects the distinct biological information captured by these metrics: TLG integrates both metabolic activity and tumor volume. Since metabolic intensity often reflects tumor aggressiveness and dedifferentiation, TLG might be more sensitive in predicting early systemic dissemination. Conversely, MTV represents the total metabolically active tumor burden and may therefore better represent overall disease bulk that drives long-term outcomes such as survival. In addition to these, low baseline MTV (2.0) appeared to partially mitigate the adverse prognostic effect traditionally attributed to unfavorable histologic response. However, these findings should be considered hypothesis-generating rather than practice-changing. Larger prospective studies are needed to validate these observations.

## Electronic Supplementary Material

Below is the link to the electronic supplementary material.

Supplementary Material 1
