# Supplementary material for: 18 F-FDG PET/CT Derived Volumetric Parameters Can Predict Outcome in Pediatric Ewing Sarcoma
Source: Ann Nucl Med. 2026 May 21;40(9):1121–30. doi: 10.1007/s12149-026-02229-6 (PMC13506628; doi:10.1007/s12149-026-02229-6)
Supplement: Supplementary file 1 [file 12149_2026_2229_MOESM1_ESM.docx]

**Supplementary Table 1** Treatment protocols

| **Treatment Component** | **n** | **Details** |
| --- | --- | --- |
| **Interval between PET1 and neoadjuvant chemotherapy** |  | median: 2 days |
| **Neoadjuvant chemotherapy regimens including all patients*** |  |  |
| VIDE (vincristine, ifosfamide, doxorubicine, etoposide) | 42 | 38 completed 6 courses; 4 completed 4 courses |
| PIV (cisplatin, ifosfamide, vincristine) | 9 | 6 completed 1 course; 3 completed 2 courses |
| VDC-P (vincristine, doxorubicin, cyclophosphamide, cisplatin) | 3 | all received 4 courses |
| VDC-IE | 1 | 4 alternating courses |
| **Chemotherapy regimens between PET1 and PET2** |  | 24 patients had PET2 |
| VIDE | 13 | 9 received 6 courses; 4 received 3 courses |
| PIV | 8 | 7 received 1 course; 1 received 2 courses |
| VDC-P | 3 | all received 4 courses |
| **Interval between chemotherapy and PET2** |  | median: 24 days |
| **Additional chemotherapy between PET2 and local control** |  |  |
| VIDE | 4 | 3 courses |
| PIV | 1 | 1 course |
| **Interval between PET2 to surgery** |  | median: 25 days |
| **Local control** |  |  |
| Surgical resection | 46 | performed median 35 days after neoadjuvant chemotherapy in overall cohort |
| Postoperative radiotherapy | 23 | 14 received to primary site; 4 to metastatic sites; 5 to both. |
| Radiotherapy alone (no surgery) | 5 |  |
| Surgery not performed due to family refusal | 2 | 1 received radiotherapy |
| Surgery not performed due to progression | 1 |  |
| Lost to follow-up after neoadjuvant chemotherapy | 1 |  |

*Treatments were administered according to the institutional protocols based on risk stratification.

PET1: baseline 18F-FDG PET/CT, PET2: 18F-FDG PET/CT after neoadjuvant chemotherapy.

**Supplementary Table 2** Comparison of PET parameters according to histopathological response

| **Variables** | **n** | **Favorable response** | **n** | **Unfavorable response** | **p** |
| --- | --- | --- | --- | --- | --- |
| **SUVmax** |  |  |  |  |  |
| PET1 | 30 | 6.1 (1.6-22.7) | 10 | 4.3 (1.9-19.5) | 0.617 |
| PET2 | 14 | 1.8 (0.6-3.3) | 4 | 2.4 (1.3-3.3) | 0.339 |
| ∆PET | 14 | -62% | 4 | -57% | 0.595 |
| **SUVpeak** |  |  |  |  |  |
| PET1 | 30 | 4.7 (1.3-18.7) | 10 | 3.4 (1.6-17.3) | 0.595 |
| PET2 | 14 | 1.3 (0.5-2.8) | 4 | 2.1 (0.8-2.6) | 0.524 |
| ∆PET | 14 | -65% | 4 | -61% | 0.595 |
| **MTV (40%)** |  |  |  |  |  |
| PET1 | 30 | 77.2 (6.4-1264) | 10 | 65.4 (12.9-476) | 0.888 |
| PET2 | 14 | 33.5 (1.6-128) | 4 | 25.8 (10.3-120) | 0.595 |
| ∆PET | 14 | -67% | 4 | -28% | 0.243 |
| **MTV (45%)** |  |  |  |  |  |
| PET1 | 30 | 63.2 (5.2-956.7) | 10 | 53.5 (10.4-370) | 0.975 |
| PET2 | 14 | 28.1 (1.4-125) | 4 | 16.2 (6.7-82.4) | 0.457 |
| ∆PET | 14 | -71% | 4 | -45% | 0.396 |
| **MTV (2.0)** |  |  |  |  |  |
| PET1 | 30 | 55.7 (0-1824) | 10 | 48.7 (0-1030) | 0.925 |
| PET2 | 14 | 0 (0-11.8) | 4 | 1.7 (0-3.2) | 0.221 |
| ∆PET | 12* | -100% | 4 | -99% | 0.218 |
| **MTV (2.5)** |  |  |  |  |  |
| PET1 | 30 | 41.4 (0-1665) | 10 | 21.5 (0-970) | 0.938 |
| PET2 | 14 | 0 (0-4.6) | 4 | 0.1 (0-2.2) | 0.527 |
| ∆PET | 12* | -100% | 4 | -99% | 0.486 |
| **TLG (40%)** |  |  |  |  |  |
| PET1 | 30 | 236.1 (23.1-7324.7) | 10 | 153.2 (22.6-4240.8) | 0.755 |
| PET2 | 14 | 42.5 (1.4-133) | 4 | 40.4 (7.5-1256) | 0.915 |
| ∆PET | 14 | -89% | 4 | -73% | 0.396 |
| **TLG (45%)** |  |  |  |  |  |
| PET1 | 30 | 179.1 (19.9-6364.5) | 10 | 131.4 (19.6-3759) | 0.617 |
| PET2 | 14 | 39.5 (1.3-89.1) | 4 | 27.8 (5.4-115.9) | 0.750 |
| ∆PET | 14 | -92% | 4 | -73% | 0.671 |
| **TLG (2.0)** |  |  |  |  |  |
| PET1 | 30 | 193.9 (0-10205.6) | 10 | 123.6 (0-6154.9) | 0.901 |
| PET2 | 14 | 0 (0-28.9) | 4 | 3.8 (0-8.6) | 0.221 |
| ∆PET | 12* | -100% | 4 | -99% | 0.218 |
| **TLG (2.5)** |  |  |  |  |  |
| PET1 | 30 | 161.5 (0-9839) | 10 | 62.8 (0-6037.7) | 0.839 |
| PET2 | 14 | 0 (0-13) | 4 | 0.1 (0-6.5) | 0.527 |
| ∆PET | 12* | -100% | 4 | -99% | 0.486 |

Continuous variables are presented as median (min-max) values. PET1: baseline 18F-FDG PET/CT, PET2: 18F-FDG PET/CT after neoadjuvant chemotherapy, ∆PET: percentage change between PET1 and PET2, is presented as median value, MTV: metabolic tumor volume, TLG: total lesion glycolysis.

*For cases in which MTV (2.0), MTV (2.5), TLG (2.0), and TLG (2.5) values were 0 at both PET1 and PET2, the corresponding ΔMTV (2.0 and 2.5) and ΔTLG (2.0 and 2.5) could not be computed.
